# First record of *Ianiropsis* cf. *serricaudis* in Maryland Coastal Bays, USA (Crustacea, Peracarida, Janiridae)

**DOI:** 10.3897/zookeys.747.22754

**Published:** 2018-04-04

**Authors:** Andrés G Morales-Núñez, Paulinus Chigbu

**Affiliations:** 1 NSF – CREST Center for the Integrated Study of Coastal Ecosystem Processes and Dynamics in the Mid-Atlantic Region (CISCEP), Princess Anne, USA; 2 NOAA Living Marine Resources Cooperative Science Center (LMRCSC), Department of Natural Sciences, University of Maryland Eastern Shore, Princess Anne, MD 21853, USA

**Keywords:** *Ianiropsis* cf. *serricaudis*, Isopoda, Maryland Coastal Bays (MCBs), mid-Atlantic Region

## Abstract

During monthly sampling of benthic invertebrates at 13 stations in the Maryland Coastal Bays (MCBs) from March to December 2012, a total of 29 individuals of Ianiropsis cf. serricaudis were collected. This species is being reported for the first time in MCBs. A detailed illustration and description of an adult male of I. cf. serricaudis from MCBs is presented. An illustrated key of males of *Ianiropsis* species belonging to the palpalis-group is also presented. The size of the largest male was 3.0 mm and that of the largest female was 2.5 mm. It is possible that I. cf. serricaudis was present in the MCBs, but overlooked during previous surveys of marine benthic invertebrates in the area because of its small body size and lack of taxonomic expertise.

## Introduction

**Figure 1.**
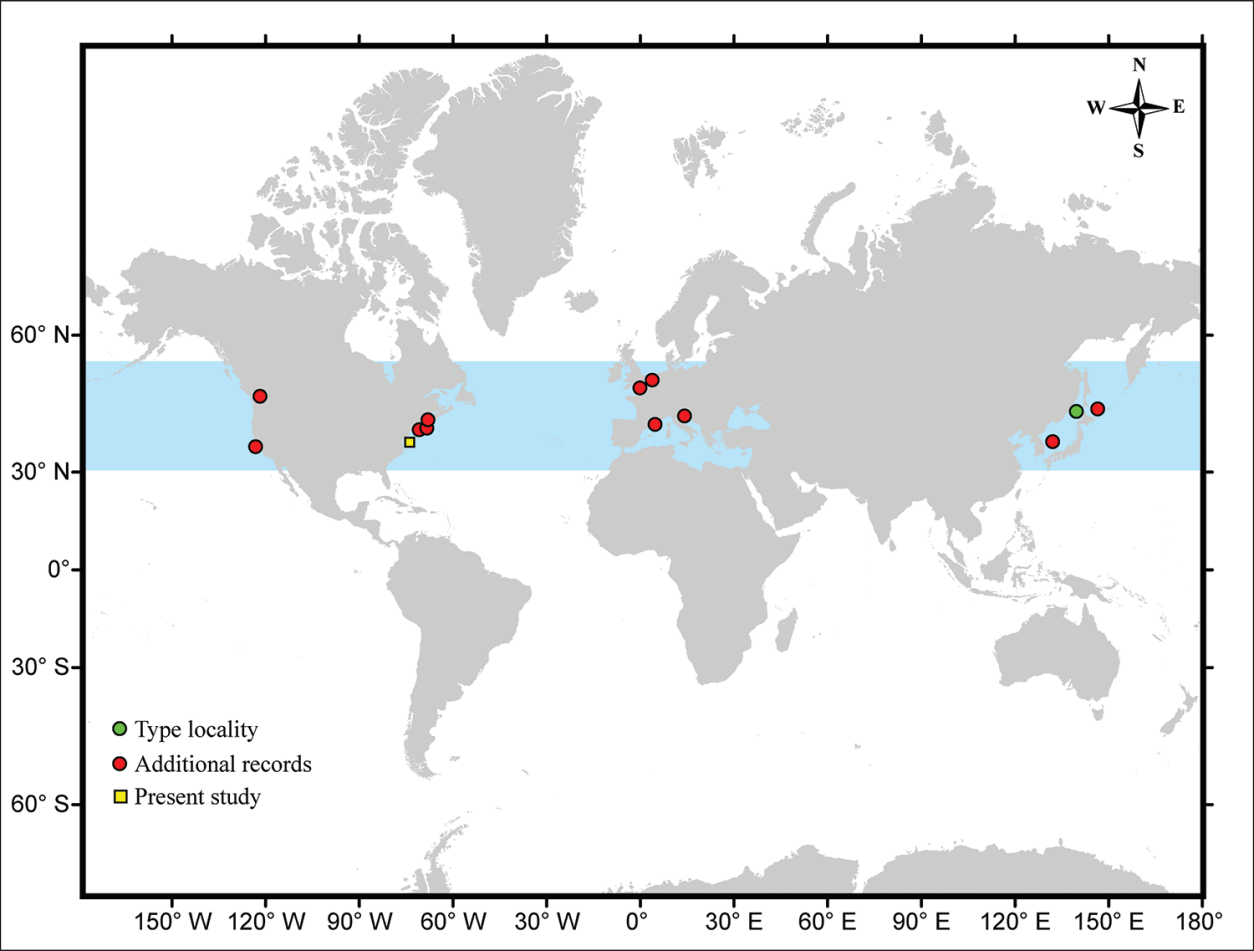
Map showing the worldwide distribution of *Ianiropsis serricaudis* and I. cf. serricaudis. *Ianiropsis serricaudis* – type material (green circle), *I. serricaudis* – additional records (red circle), I. cf. serricaudis (yellow square). Data from: Gurjanova (1936); Kussakin (1962, 1988); Jang and Kwon (1990); Hobbs et al. (2015); Marchini et al. (2016); Ulman et al. (2017); Morales-Núñez and Chigbu (this study).

*Ianiropsis serricaudis* Gurjanova, 1936 is a janirid isopod that was described from the Russian coast of the Sea of Japan (Fig. 1). Unfortunately, the original description is poor and the illustrations are incomplete (Gurjanova 1936). Specimens of *I. serricaudis* have also previously been reported in the Sea of Okhotsk, on the Coast of Iturup Island (one of the Kuril Islands) from Russia (Kussakin 1962, 1988), Korea (Jang and Kwon 1990), and recently as a successful invasive species of both coasts of the United States and Europe (Hobbs et al. 2015; Marchini et al. 2016; Ulman et al. 2017) (Fig. 1). In the coastal waters of the northeastern United States, it has only been reported from Gulf of Maine to Barnegat Bay, NJ whereas in Europe it is known only from England, Netherlands, Italy, and France (Hobbs et al. 2015; Marchini et al. 2016; Ulman et al. 2017) (Fig. 1). These authors presented additional descriptions of the body and/or appendages of specimens of the species and showed morphological differences among specimens from various geographic locations (see Kussakin 1962, 1988; Jang and Kwon 1990; Hobbs et al. 2015). Additionally, Saito et al. (2000) considered *I. notoensis* as a junior synonym of *I. serricaudis* despite exhibiting multiple differences with Gurjanova’s original description. Despite these differences, no one has revised the original description of the type material of *I. serricaudis*, or at least specimens from the precise type locality (topotypes). Without such a revision, and more detailed morphological and molecular analyses of these populations, the records attributed to this species remain uncertain.

We have tried to find the type material of *I. serricaudis*, but the exact location (i.e., museum collection) where it was placed was not indicated in the original description; it is unknown whether or not it has been lost. For this reason, Dr. Viktor Petryashov (Zoological Institute, Russian Academy of Sciences, Saint Petersburg, Russia) was contacted and asked if the type material might be located at this institute, but, regrettably, the only material available is a female collected in 1948 (V. Petryashov pers. comm. 2017).

**Figure 2.**
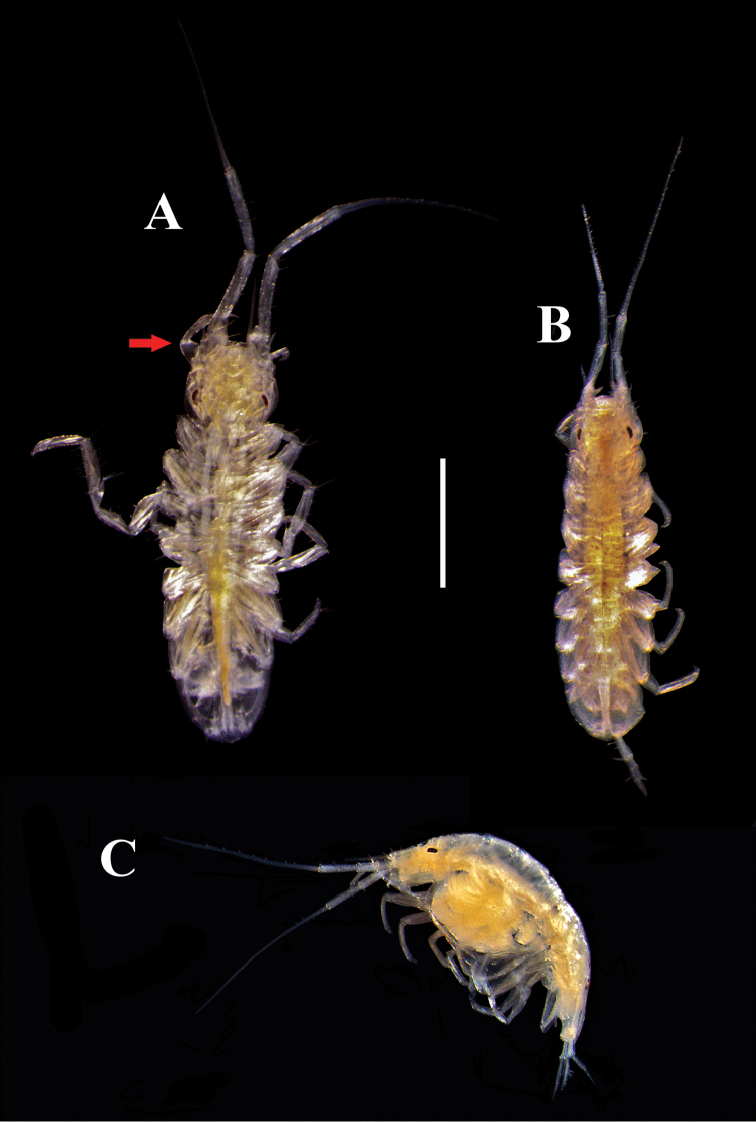
Pictures of habitus of Ianiropsis cf. serricaudis from Maryland Coastal Bays. **A** adult ♂, dorsal view, 3.0 mm TL **B** ♀ with oostegites, dorsal view, 2.7 mm TL **C** ovigerous ♀, lateral view, 2.26 mm TL. Arrow indicate the long maxillipedal palp from dorsal view on ♂. Scale bar: 1.0 mm.

During examination of monthly samples of benthos collected in 2012 from the Maryland Coastal Bays (MCBs), a number of specimens attributable to Ianiropsis cf. serricaudis were observed (Figs 1–2). This study aims to report, for the first time, the presence of I. cf. serricaudis in the MCBs. Additionally, a detailed supplementary illustration and description of an adult male of I. cf. serricaudis from MCBs is presented herein.

## Materials and methods

### Study area

**Figure 3.**
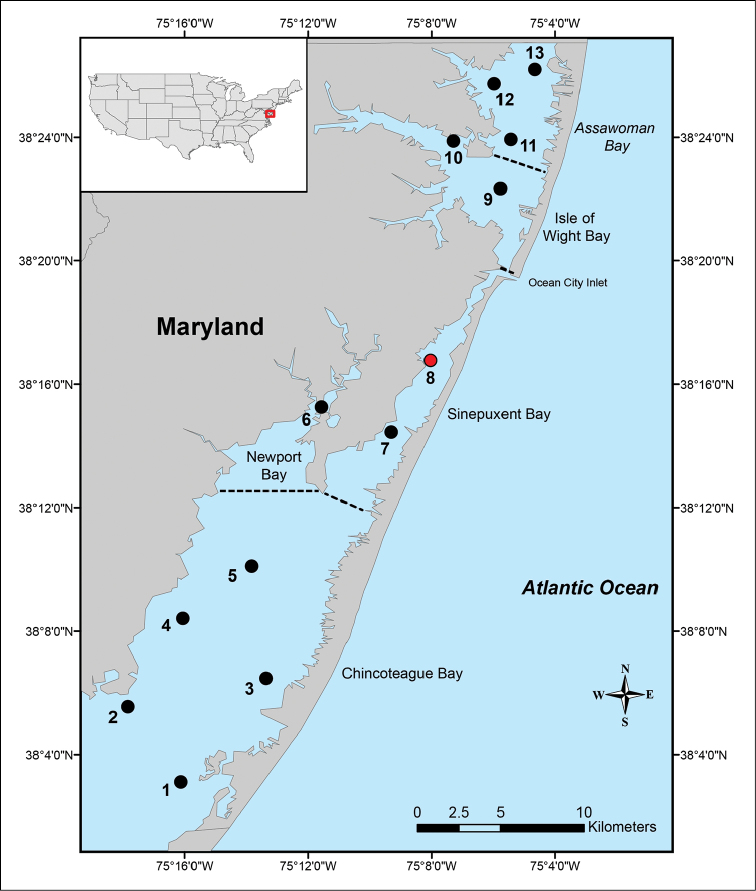
Map of Maryland Coastal Bays indicating the 13 stations sampled. Red circle (station 8) indicates the only station where Ianiropsis cf. serricaudis was collected; dotted lines separate bays.

The Maryland Coastal Bays is a barrier-island system located on the eastern part of the Delmarva Peninsula in the United States of America (USA). The system consists of five principal lagoons distributed in two areas; Assawoman and Isle of Wight Bays located in the northern area of MCBs, and Sinepuxent, Newport, and Chincoteague Bays located in the southern area of MCBs (Fig. 3). These five bays differ with regard to depth, flushing rate, surface area, and anthropogenic activity. In general, the MCBs are shallow with an average depth of 1.2 m, predominantly polyhaline with salinity greater than 25, and surface area that ranges from 15.9 km^2^ in Newport Bay to 189 km^2^ in Chincoteague Bay (Chaillou et al. 1996; Wazniak et al. 2004).

### Sample collection and processing

Samples were taken at 13 sites (eight sites in the southern area and five in the northern area) (Fig. 3). Sampling was conducted monthly for nine months from March to December 2012, although due to inclement weather conditions, samples were not collected in September. Samples were collected using an epibenthic sled (area = 0.39 m²), with a 1.0 mm mesh size net. A flow meter Model 2030R (General Oceanics) was attached to the net frame in order to determine the volume of water that passed through the net during each sampling event. Field sampling was completed in two days each month. At each site, two horizontal tows were conducted at an average speed of 2 knots for 5 min. In the field, the net was rinsed and all macroinvertebrates were passed through a 0.5 mm sieve. Additionally, epifauna were separated from macroalgae by shaking each macroalgal fragment in a bucket filled with seawater. The macroinvertebrates retained were passed through a 0.5 mm sieve and all the invertebrates were fixed in 5% neutral buffered formalin. All macroalgae collected with the sled were stored in plastic bags with seawater in a cooler. The macroalgae were washed over a sieve with a 0.3 mm mesh size. Each macroalgal fragment was then visually examined further to confirm that all epifaunal invertebrates had been removed. All invertebrates collected were counted, identified to the lowest practical taxonomic level, and preserved in ethanol (70 %).

Water quality data were collected *in situ* using a YSI 6600 Multi-Parameter Water Quality Sonde and included water temperature, salinity, dissolved oxygen, and pH, which were all recorded at 0.3 m from the bottom (Table 1). Additionally, water depth and clarity (i.e. Secchi disc transparency) were recorded at each site.

**Table 1.** Mean ± SE monthly values of abiotic variables from March to December 2012 in MCBs. * No data were collected.

Months	Temperature (°C)	Salinity (PSU)	Dissolved Oxygen (mg L^-1^)	pH	Depth (m)	Secchi Depth (m)
March	11.8 ± 0.60	32.2 ± 1.20	9.1 ± 0.19	8.0 ± 0.05	*	*
April	14.7 ± 0.77	34.6 ± 0.76	7.9 ± 0.10	8.0 ± 0.02	1.9 ± 0.13	*
May	17.2 ± 0.49	34.1 ± 0.53	7.6 ± 0.19	8.0 ± 0.02	1.6 ± 0.26	0.7 ± 0.10
June	21.7 ± 0.34	32.5 ± 0.46	6.9 ± 0.13	7.8 ± 0.40	1.9 ± 0.14	0.7 ± 0.06
July	26.4 ± 0.23	32.9 ± 0.45	6.0 ± 0.16	7.8 ± 0.03	2.0 ± 0.13	0.6 ± 0.04
August	24.5 ± 0.11	34.4 ± 0.51	5.7 ± 0.24	7.7 ± 0.04	1.7 ± 0.21	*
October	17.6 ± 0.1	27.6 ± 0.6	8.1 ± 0.1	8.0 ± 0.0	2.1 ± 0.16	1.2 ± 0.06
November	9.6 ± 0.2	26.4 ± 0.8	10.4 ± 0.3	8.0 ± 0.0	2.2 ± 0.26	1.0 ± 0.10
December	8.0 ± 0.1	28.2 ± 0.6	10.3 ± 0.1	8.0 ± 0.0	2.1 ± 0.24	1.0 ± 0.20

Specimens of I. cf. serricaudis were dissected under an Olympus SXZ16 stereomicroscope. Appendages were mounted on glass slides in glycerin and observed with an Olympus BX41 compound microscope, and drawings were made with a *camera lucida*. Illustrations were prepared with Adobe Illustrator CS6 Extended. Photographs were taken using an Olympus DP73 digital camera mounted on a stereomicroscope Olympus SXZ16 and all specimens were measured with CellSens dimensions 1.11 software (Olympus). Specimens have been deposited in the National Museum of Natural History, Smithsonian Institution, Washington, DC (USNM). All measurements are in millimeters (mm). Total body length (TL) was measured from the frontal margin of the head to the tip of the pleotelson.

All specimens of Ianiropsis cf. serricaudis were sexed and classified into three categories:

(1) males (Fig. 2a) with maxillipedal palp visible on dorsal view, nonetheless, when the maxillipedal palp could not be observed in dorsal view, the triangular shape at the apex of the pleopod-1 was used, (2) females with oostegites (Fig. 2b), and (3) ovigerous females (Fig. 2c) with embryos in the marsupium (Fig. 2c).

We were not able to re-examine the type material or topotypes of *Ianiropsis serricaudis* in order to clarify uncertainties about the morphology of this species. For this reason, identification of the specimens from this study is based on the morphological characters from previous descriptions (Gurjanova 1936; Kussakin 1962, 1988; Jang and Kwon 1990; Hobbs et al. 2015).

Nevertheless, specimens of *Ianiropsis* sp. from Florida and off Virginia coast logged at the USNM were examined to determine whether they are conspecific with *I. serricaudis*.

### Abbreviations

**MCBs** Maryland Coastal Bays

**PSU** Practical Salinity Unit

**USNM** National Museum of Natural History, Smithsonian Institution, Washington DC

**TL** Total body length

## Results

### Abiotic variables

The mean (±SE) values of environmental parameters measured in the MCBs during this study period are summarized in Table 1. Mean temperature (°C) ranged from 8.0 ± 0.1 to 26.4 ± 0.23, salinity (PSU) from 26.4 ± 0.8 to 34.6 ± 0.76, and dissolved oxygen (mg l^–1^) from 6.0 ± 0.16 to 10.4 ± 0.3. Furthermore, pH ranged from 7.7 ± 0.04 to 8.0 ± 0.05, depth (m) from 1.7 ± 0.21 to 2.2 ± 0.26, and Secchi depth (m) transparency from 0.6 ± 0.04 to 1.2 ± 0.06.

### Composition of population

**Figure 4.**
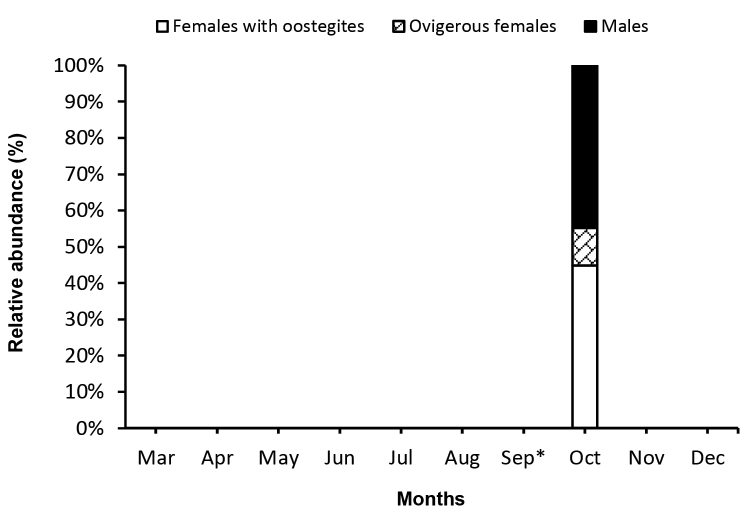
Relative abundance of developmental stages of I. cf. serricaudis in 2012. *Samples were not taken in September.

A total of 29 individuals of Ianiropsis cf. serricaudis was counted and sexed. Among them, 13 were females with oostegites, three were ovigerous females, and 13 were males (Table 2). Specimens of I. cf. serricaudis were only found in one of thirteen stations along the bays (Fig. 2); all of them were found in October (2012) (Fig. 4).

### Size-distribution of population

The body sizes of individual Ianiropsis cf. serricaudis measured in the MCBs during this study are presented in Table 2. Females with oostegites varied from 0.95 to 2.52 mm; mean TL was 1.98 ± 0.15 (*n* = 12). The smallest observed ovigerous female was 1.91 mm, while the largest was 2.34 mm, mean TL was 2.17 ± 0.13 (*n* = 3). Males ranged from 1.38 mm to 3.0 mm; mean TL was 2.21 ± 0.13 (*n* = 13).

**Table 2.** TL and comparison of morphological features of stages of Ianiropsis cf. serricaudis from Maryland Coastal Bays.

Stages	TL (mm)	No. of antennular articles (Left–Right)	Length–Antenna (mm)	Length antennal articles 5–6 (Left–Right)	No. of lateral spines of pleotelson (Left–Right)	Maxilliped–dorsal view
**Females with oostegites**						
1	0.99	7–7	1.0	0.23–0.22	3–3	
2	0.95	7–7	1.0	0.21–0.23	3–3	
3	1.98	10–10	Missing	Missing	4–4	Not visible
4	2.26	11–11	1.68	0.60–missing	2–2	Not visible
5	1.95	10–10	1.86	0.48–0.45	4–4	Not visible
6	2.52	9–10	2.22	0.73–0.57	4–3	Not visible
7	2.44	12–12	2.09 (broken)	0.77–missing	4–2?	Not visible
8	2.36	11–11	2.28	0.67–0.71	4–4	Not visible
9	2.33	11–11	2.28	0.57–0.61	3–4	Not visible
10	2.21	10–11	Missing	Missing	4–2?	Not visible
11	1.89	Missing	Missing	Missing	3–3	Not visible
12	1.86	10–10	2.23	Missing–0.47	3–4	Not visible
13	Broken	12–12	Missing	Missing	Missing	Not visible
Mean ± SE	1.98 ± 0.15					
**Ovigerous females**						
1	1.91	10–10	Missing	Missing	3–4	Not visible
2	2.26	10–10	2.22	0.57–0.56	3–3	Not visible
3	2.34	11–11	2.39	Missing–0.60	2–2	Not visible
Mean ± SE	2.17 ± 0.13					
**Males**						
1	2.14	Damaged	2.45	0.76–0.78	4–3	Not visible
2	2.2	11–12	2.39	0.64–0.65	3–3	Not visible
3	3.0	13–14	3.02	1.21–1.17	4–4	Visible
4	1.76	10–10	Missing	Missing	3–3	Not visible
5	1.53	Missing–10	1.12	0.31–missing	4–4	Not visible
6	1.38	9–9	1.4	0.34–0.37	3–3	Not visible
7	2.43	13–13	3.0	1.02–0.76	3–3	Visible
8	2.7	14–13	1.20 (broken)	0.36–missing	3–4	Visible
9	2.93	12–13	3.1	1.29–1.24	4–4	Visible
10	2.22	12–12	Missing	Missing	3–3	Visible
11	2.19	12–12	1.59 (broken)	0.63–0.49	4–4	Not visible
12	2.17	12–12	1.49 (broken)	Missing–0.76	4–4	Visible
13	2.09	11–11	2.28	0.61–0.58	4–2	Not visible
Mean ± SE	2.21 ± 0.13					

## Systematics

### Order Isopoda Latreille, 1817

#### Suborder Asellota Latreille, 1802

##### Superfamily Janiroidea Sars, 1897

###### Family Janiridae Sars, 1897

####### Genus *Ianiropsis* Sars, 1897

######## Ianiropsis cf. serricaudis

Taxon classificationAnimaliaIsopodaJaniridae

Gurjanova, 1936

Figures 2 , 5 , 6 , 7 , 8 , 9 , 10 , 11 , 12 , 13D

######### Material examined.

13 ♂♂ (three USMN: 1480972, 1480973, and 1480974), one ovigerous ♀, two ♀♀ carrying juveniles (one USNM 1480975), and 13 ♀♀ with oostegites (two USNM: 1480976 and 1480977), station-8 (38°16.825'N − 75°08.032'W), Sinepuxent Bay, USA, depth 3.1 m, October 25-2012, collected by A.G. Morales-Núñez.

######### Description.

**Figure 5.**
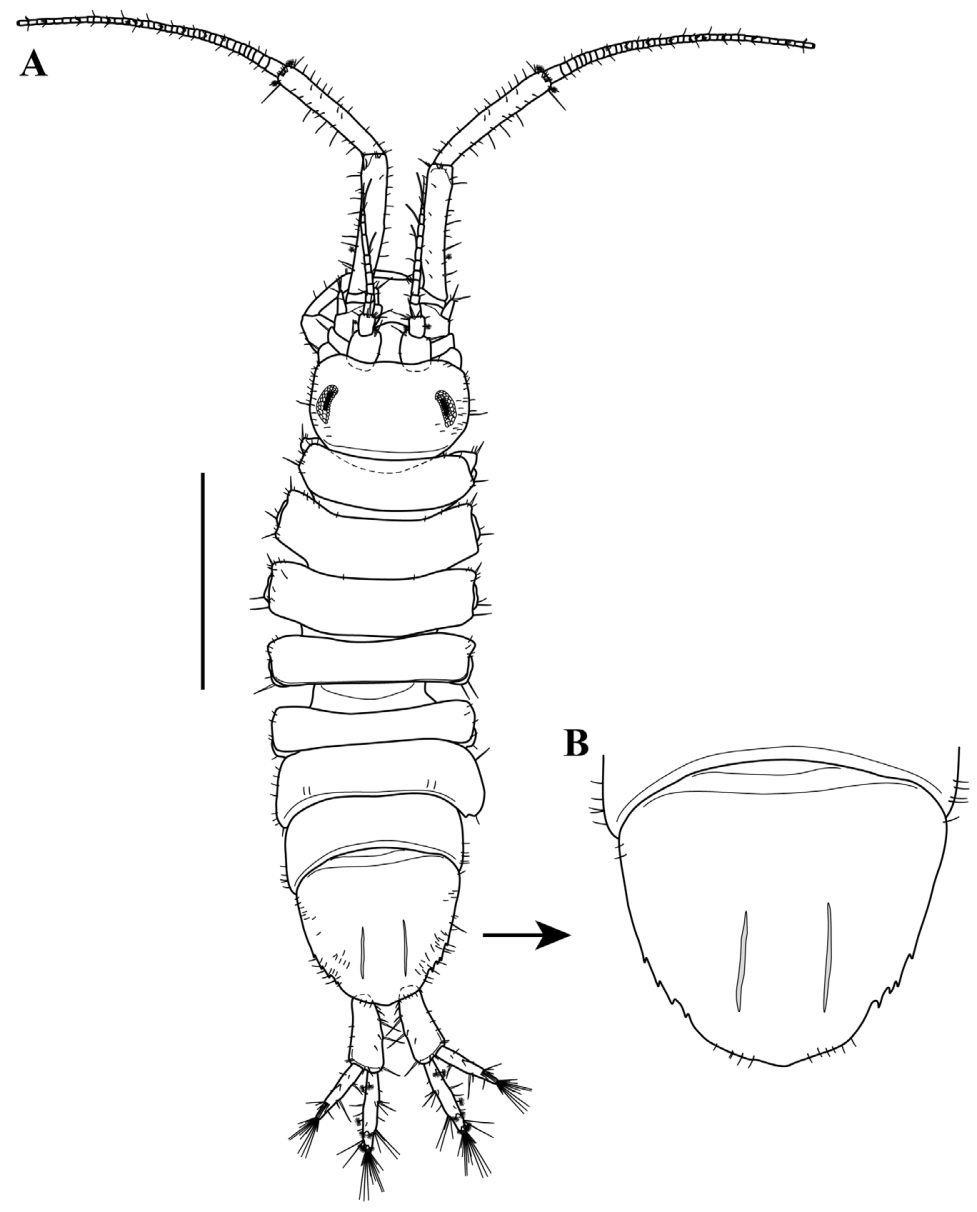
Ianiropsis cf. serricaudis from MCBs, Adult ♂. **A** dorsal view **B** enlargement of pleotelson. Scale bar: 1.0 mm (**A**).

Based on adult terminal ♂ of Ianiropsis cf. serricaudis from Maryland Coastal Bays. *Body* (Fig. 5A). TL 3.0 mm, about 3.1 times as long as wide, pigmentation in preservative scattered brown pigment.

*Head* (Fig. 5A). ~0.15 TL, 1.7 width, anterior margin with a light median convexity, posterior margin linear, each lateral margin with small simple setae of various lengths, longer than pereonite 1. Eyes dorsal, set back from lateral margin, pigmented with more than 15 well-developed ommatidia.

*Pereon* (Fig. 5A). ~0.6 TL, all pereonites wider than long, pereonites 1–3 sub-equal in length, pereonites 4 and 5 shorter than pereonites 1–3 and 5–6, both lateral margins of each pereonite with small simple setae of various lengths.

*Pleotelson* (Fig. 5A–B). ~0.25 TL, ovate, with three to four denticles on each lateral margin (Fig. 5A–B).

**Figure 6.**
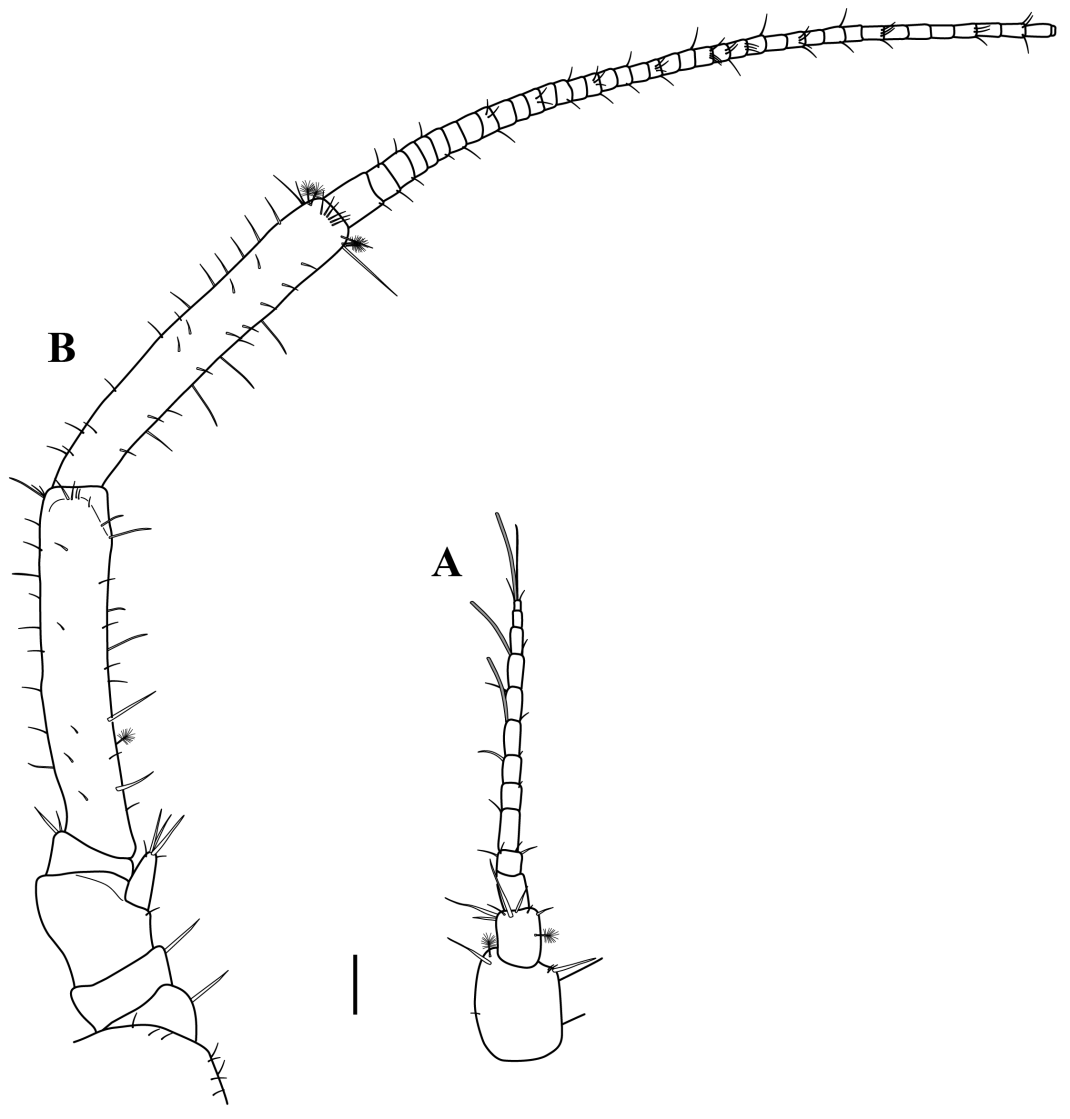
Ianiropsis cf. serricaudis from MCBs, Adult ♂. **A** antennule **B** antenna. Scale bar: 0.1 mm.

*Antennule* (Figs 5A, 6A). ~0.25 TL, tip reaching 0.73 of length of antennal article 5, with 13–14 articles. Article 1 widest, 1.1 times as long as wide, inner margin with a sub-proximal small simple seta and two (one simple and one sensory) distal setae; outer margin with three (two short) distal setae. Article 2, 1.3 times as long as wide, distal margin with row of seven simple setae of unequal lengths; outer mid-margin with sensory seta. Article 3, 1.2 times as long as wide, asetose. Flagellum with ten articles; articles 8, 10 and 13 with one aesthetasc. Article 13 minute, with three distal simple setae of various lengths.

*Antenna* (Figs 5A, 6B). As long as body, articles 1–6 about ½ of TL. Articles 1 and 2, wider than long, with simple seta on distal outer margin. Article 3 sub-quadrate, with small seta near to the insertion of the antennal scale. Article 4, wider than long, with two simple setae on inner distal margin. Article 5, twice as long as combined lengths of articles 1–4. Article 6, slightly shorter than article-5. Flagellum with 39–45 sub-equal articles. Antennal scale longer than article 4, with five simple setae on distal margin of varying lengths.

**Figure 7.**
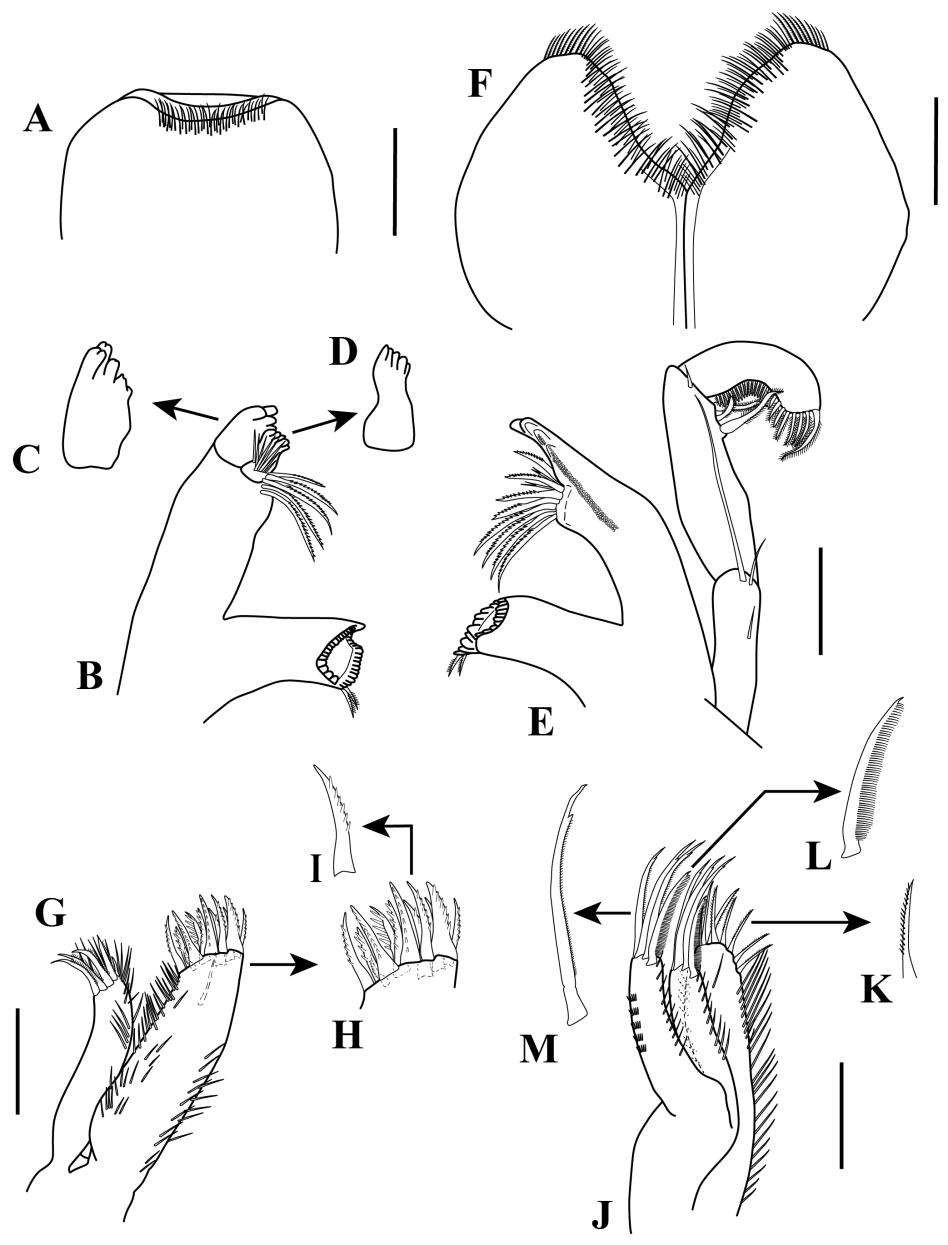
Ianiropsis cf. serricaudis from MCBs, Adult ♂. **A** labrum **B** left mandible **C** enlargement of incisor process **D** enlargement of *lacinia mobilis* **E** right mandible **F** lower lip **G** maxillule **H** enlargement of tip of outer lobe **I** denticulate seta **J** maxilla **K** denticulate setae **L** enlargement of “comb-like” setae **M** enlargement of finely setulate seta. Scale bars: 0.1 mm (**A–B, E, F, G, J**).

*Mouthparts*. *Upper lip* (Fig. 7A) broad, with fine apical setae.

*Mandibles* (Fig. 7B–E). Molar process well developed with two denticulate setae (Fig. 7B and 7E, respectively); left mandible with strong incisor bearing five teeth (Fig. 7C), *lacinia mobilis* with five teeth and two rows of simple setae in the middle area (Fig. 7B, D); setal row with five denticulate setae (Fig. 7B); right mandible with incisor bearing five teeth, setal row with eight denticulate setae and two bifid setae (Fig. 7E). Palp article 1, 3.5 times as long as wide, with small simple seta on mid-margin and two (one longer and one small) simple setae on distal margin; article 2 longest, 2.9 times as long as wide, with three (two long and one short) denticulate setae on sub-distal lateral margin, with small simple seta on distal margin; article 3, 3.9 times as long as wide, with row of ± 25 denticulate setae along the lateral margin (Fig. 7E).

*Lower lip* (Fig. 7F). Two pairs of lobes with inner margins setulate.

*Maxillule* (Fig. 7G–I). Inner lobe with four setulate distal setae, outer and distal margin with simple setae. Outer lobe with 12–13 robust denticulate distal setae (Fig. 7H–I), with one simple seta on mid sub-distal margin, both margins finely setose (Fig. 7G).

*Maxilla* (Fig. 7J–M). Inner lobe with seven denticulate setae (Fig. 7K), both margins finely setose. Outer and middle lobes having one “comb-like” seta (Fig. 7L) and three finely setulate setae (Fig. 7M), both margins finely setose.

**Figure 8.**
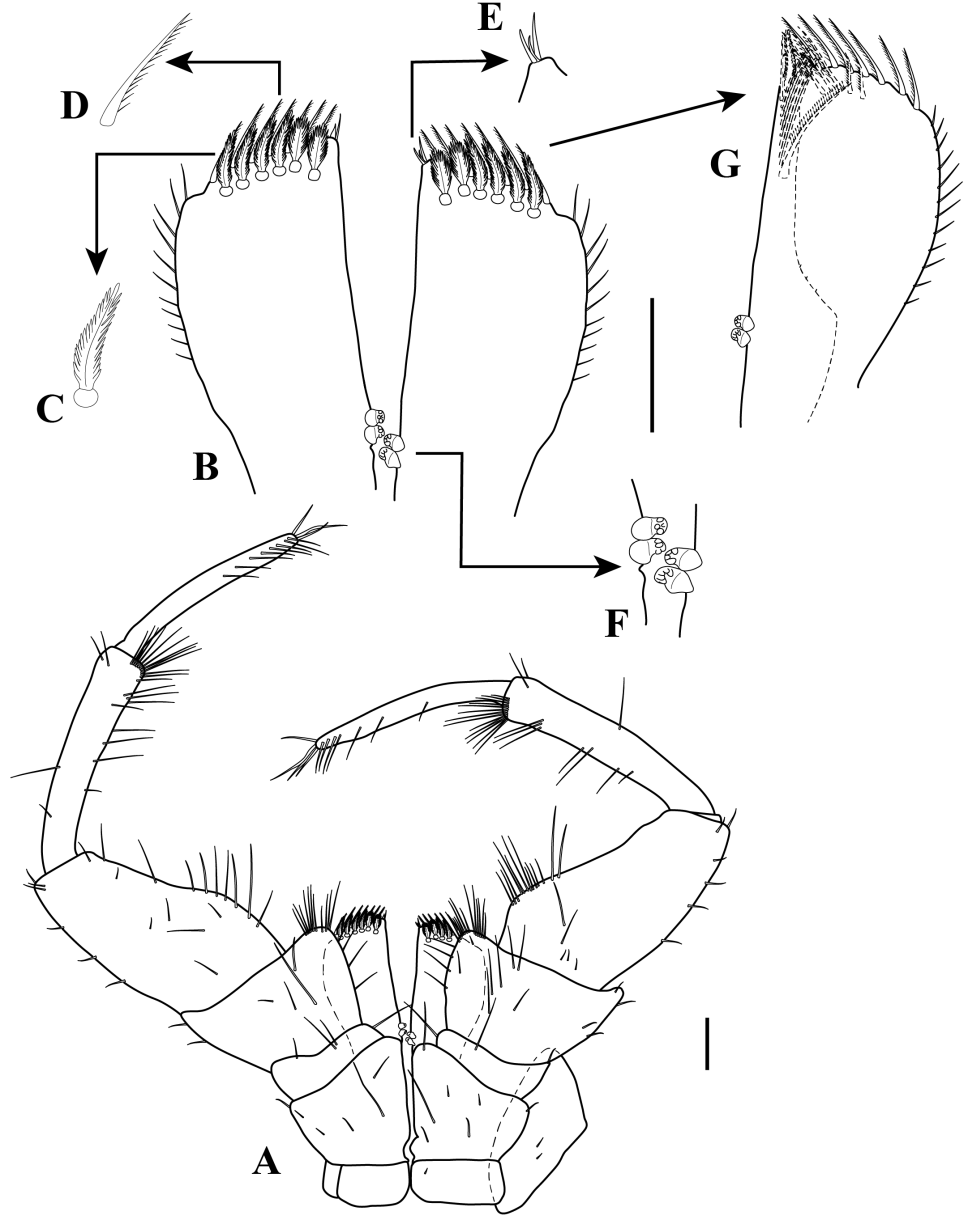
Ianiropsis cf. serricaudis from MCBs, Adult ♂. **A** maxilliped **B** endite **C** enlargement of fan setae **D** enlargement of setulate setae **E** enlargement of the distal inner margin of endite **G** detail of endite sub-distal end **F** enlargement of coupling hooks. Scale bars: 0.1 mm (**A–B, G**).

*Maxilliped* (Fig. 8A–G). Basis, 1.2 times wider than long. Endite, 2.0 times as long as wide, outer margin with 11–12 simple setae (Fig. 8B); inner proximal margin with two coupling hooks (Fig. 8B, F–G), sub-distal inner margin with one setulate seta and ~seven simple setae, sub-distal margin with seven (six on dorsal view (Fig. 8B) and one on ventral view (Fig. 8G), respectively) fan setae (Fig. 8C), distal margin with 12 (seven (Fig. 8B) and five (Fig. 8G), respectively) setulate setae (Fig. 8D), inner distal margin with three simple setae (Fig. 8E). Palp 7.4 times longer than basis (Fig. 8A): article 1, wider than long; article 2, wider than long, 3.4 times as long as article-1, inner distal margin with two clusters of simple setae of varying lengths; article 3, longer than wide, 1.7 times as long as wide, slightly longer than twice of article 2; mid-proximal inner margin greatest wide, with a row of simple setae of varying lengths; article 4 longest, 5.6 times as long as wide, with row of 12–13 simple setae on inner distal margin; article 5 sub-equal length of that of article 3, 9.5 times as long as wide, with a row of 10–11 simple setae on inner margin.

**Figure 9.**
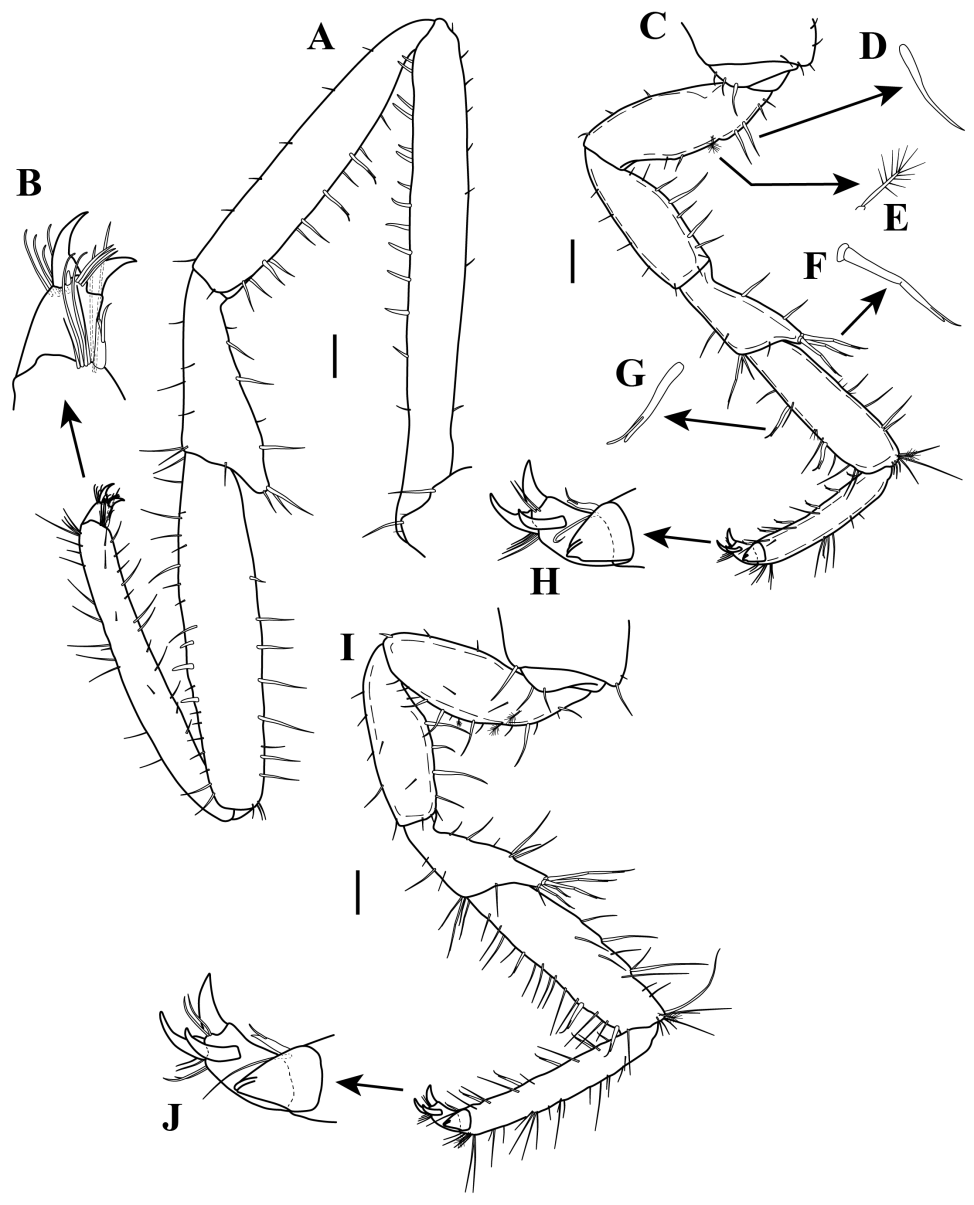
Ianiropsis cf. serricaudis from MCBs, Adult ♂. **A–B** pereopod I **C**–**H** pereopod II **I–J** pereopod III. Scale bars: 0.1 mm (**A, C, I**).

*Pereopod I* (Fig. 9A–B). Extremely longer, 1.2 times longer than TL, basis to propodus longer than other six pereopods. Basis elongate, 8.3 times as long as wide; with eight small simple setae along dorsal margin; with one simple seta and 13 robust setae along ventral margin. Ischium elongate, 5.6 times as long as wide; with 11 simple setae along dorsal margin; with one simple seta on disto-medial margin; with six simple small setae along ventral margin. Merus elongate, 2.7 times as long as wide, widest distally; with five simple setae including two small setae along dorsal margin, disto-dorsal lobe with three distal setae; with simple seta on disto-lateral margin; with six simple setae of varying lengths on ventral margin. Carpus elongate, 4.6 times as long as wide; with eight (one small) simple setae along dorsal margin and three small disto-dorsal simple setae; with 18 simple setae and three robust setae along ventral margin. Propodus elongate, 8.0 times as long as wide; with 11 (two small) simple setae along dorsal margin and cluster of four simple setae on sub-distal dorsal margin; with four simple setae on lateral margin; with 12 simple setae along ventral margin, with five simple setae of varying lengths and one robust seta on disto-ventral margin (Fig. 9B); articular plate absent. Dactylus with two distal claws, with three simple setae on disto-medial margin between the claws, with four simple setae on disto-dorsal margin (Fig. 9B).

*Pereopod II* (Fig. 9C–H). Basis, 2.4 times as long as wide; with six simple setae of varying lengths (Fig. 9D), and one sensory seta (Fig. 9E); with three small simple setae along ventral margin. Ischium, 2.6 times as long as wide; with eight simple setae along dorsal margin; with one small simple seta on distal lateral margin; with five simple setae along ventral margin. Merus, 2.0 times as long as wide, widest distally; with two simple setae along dorsal margin, disto-dorsal lobe with three robust setae (Fig. 9F); with simple setae on disto-lateral margin; with five (three distally) simple setae on ventral margin. Carpus, 3.2 times as long as wide; with seven simple setae along dorsal margin and a cluster of one sensory and six simple setae on disto-dorsal margin; with two robust setae (Fig. 9G) and ten simple setae of varying lengths along ventral margin. Propodus, 5.3 times as long as wide, with six simple setae of varying lengths and one robust seta along dorsal margin and one sensory seta and eight simple setae of varying lengths on disto-dorsal margin (Fig. 9C); with six robust setae and five simple setae along ventral margin; articular plate present on disto-lateral margin. Dactylus with three (one mid-lateral and two distal) claws, with two simple setae on distal lateral margin between the claws, with four simple setae on disto-dorsal margin (Fig. 9H).

**Figure 10.**
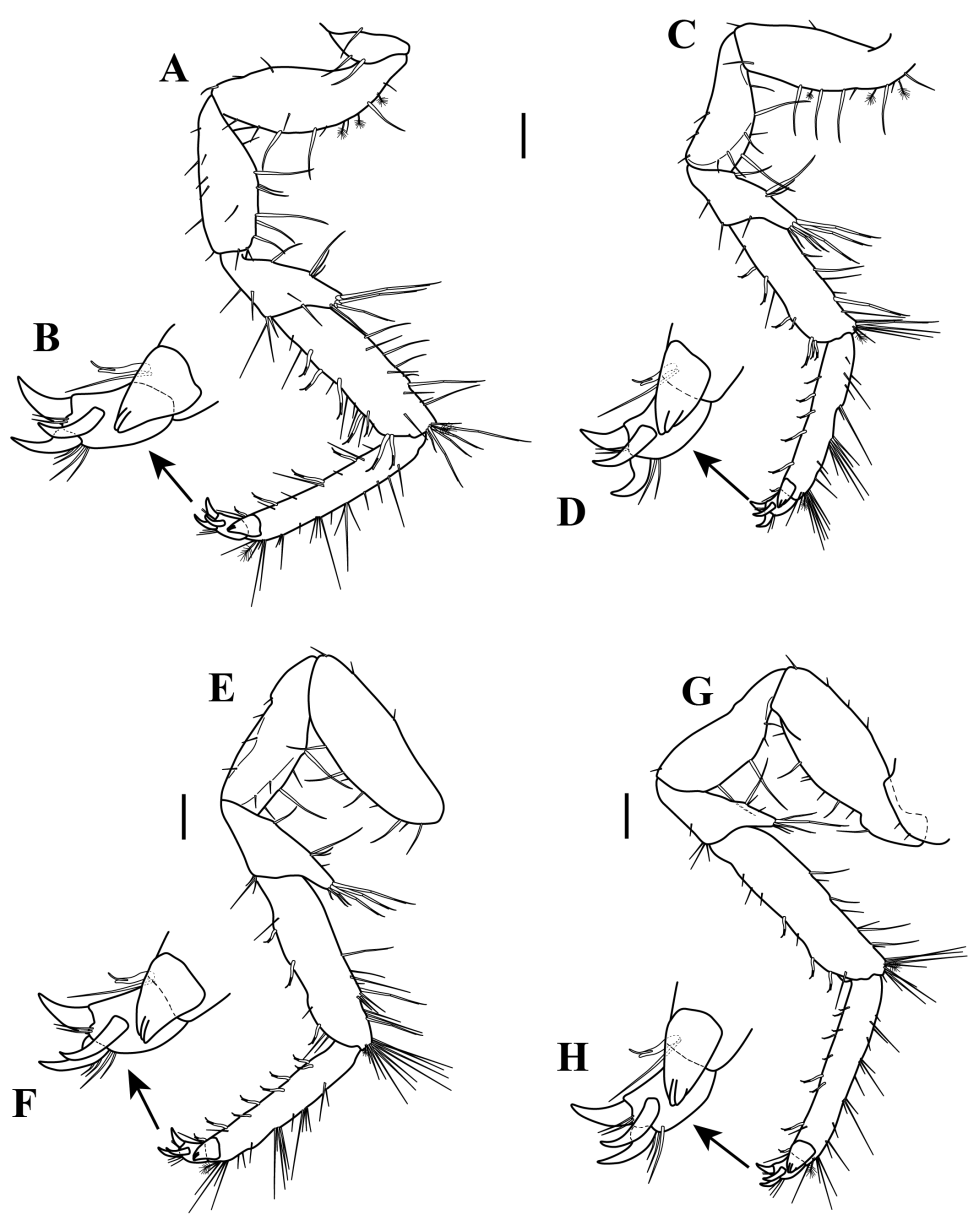
Ianiropsis cf. serricaudis from MCBs, Adult ♂. **A–B** pereopod IV **C–D** pereopod V **E–F** pereopod VI **G–H** pereopod VII. Scale bars: 0.1 mm (**A, C, E, G**).

*Pereopods III–VII* (Figs 9I–J; 10A–H, respectively). *Pereopod III* (Fig. 9I–J): 0.54 times as long as pereopod I. *Pereopod IV* (Fig. 10A–B): 0.46 times as long as pereopod I. *Pereopod V* (Fig. 10C–D): Shortest, 0.39 times as long as pereopod I. *Pereopod VI* (Fig. 10E–F): 0.45 times as long as pereopod I. *Pereopod VII* (Fig. 10G–H): 0.49 times as long as pereopod I.

**Figure 11.**
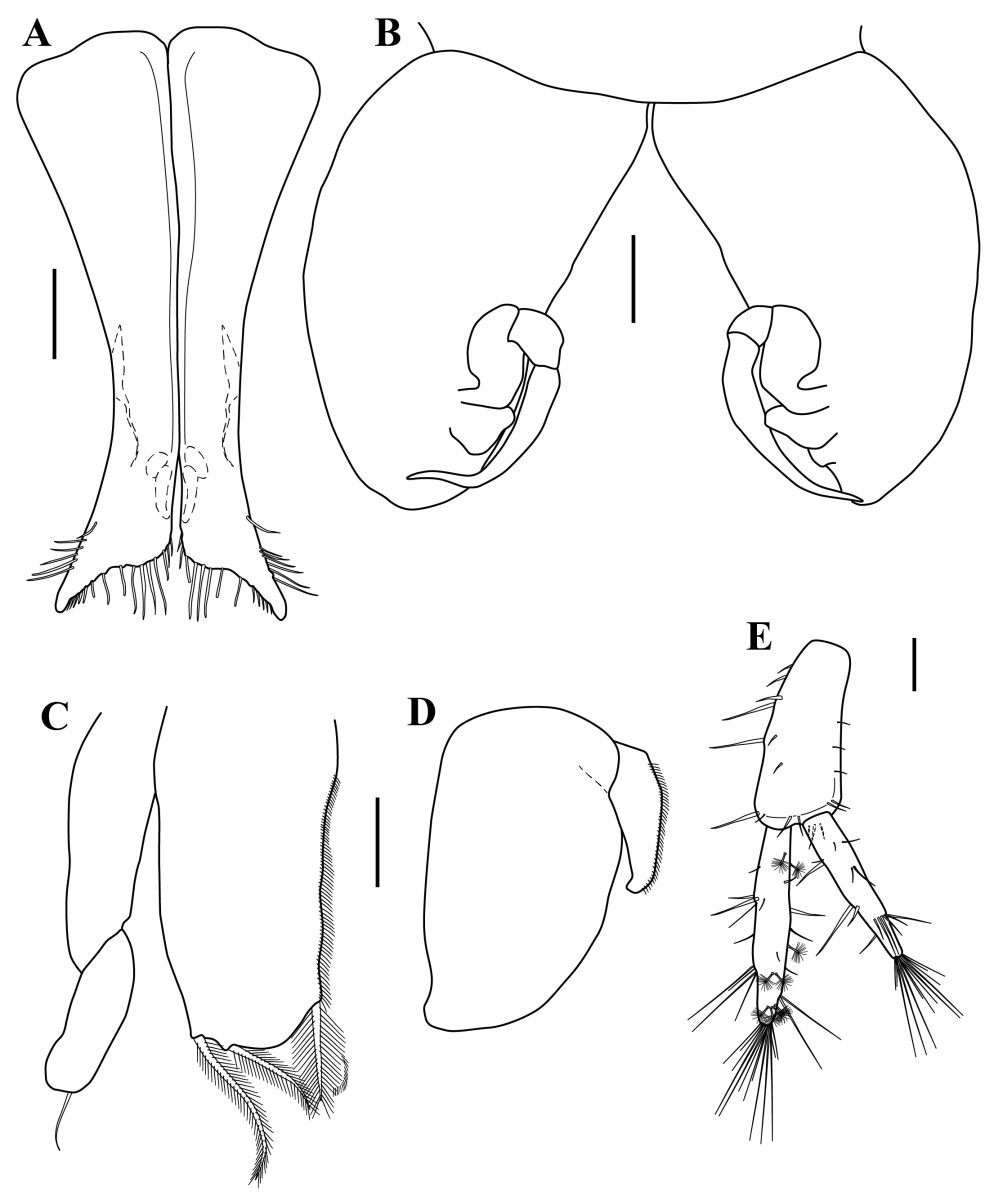
Ianiropsis cf. serricaudis from MCBs, Adult ♂. **A** pleopod I **B** pleopod II **C** pleopod III **D** pleopod IV. Scale bars: 0.1 mm.

*Pleopod I* (Fig. 11A). 8.3 times as long as wide, lateral apex pointed and directed obliquely backward, distal margin with 13–15 simple setae of unequal lengths, sub-distal outer margin with 6–7 simple setae.

*Pleopod II* (Fig. 11B). Protopod, 1.8 times as long as wide, robust, long-oval asetose, distal margin setae absent. Endopod, stylet bi-articulated, distal tip narrow, curved inwards; exopod distal margin convex, asetose.

*Pleopod III* (Fig. 11C). Endopod, 2.0 times as long as wide, distal margin rounded, inner margin setulate with plumose seta distally, outer distal margin with two plumose setae; exopod 1.2 times as long as endopod, bi-articulated, articulation between two articles oblique, article 2 distal margin broadly rounded, with simple seta.

*Pleopod IV* (Fig. 11D). Endopod, 1.7 times as long as wide, asetose; exopod 2.4 times as long as wide, reduced, 0.27 times as long as endopod, half as long as endopod, with outer margin setulate.

*Uropod* (Figs 5A, 11E). 1.1 times as long as pleotelson, slightly longer than pleotelson. Protopod 1.8 times as long as wide, 1.2 times as long as exopod and slightly shorter than endopod, with six spiniform and three simple setae along the inner margin, with two simple setae on distal margin, with five simple setae along outer margin. Endopod, 5.7 times as long as wide, longer than exopod, with several plumose, spiniform and simple setae along both margins, distal margin with 13 simple setae of varying lengths. Exopod, 5.5 times as long as wide, with spiniform and simple setae along both margins, distal margin with 13 simple setae of varying lengths.

######### Females with oostegites.

Smaller than males, mean TL 1.98 mm; TL ranges from 0.95 to 2.52 mm (Table 2). Antennule with 7–12 articles (Table 2). Antenna generally shorter than TL (Table 2); lengths of antennal articles 5–6 combined, shorter than half TL (Table 2).

**Figure 12.**
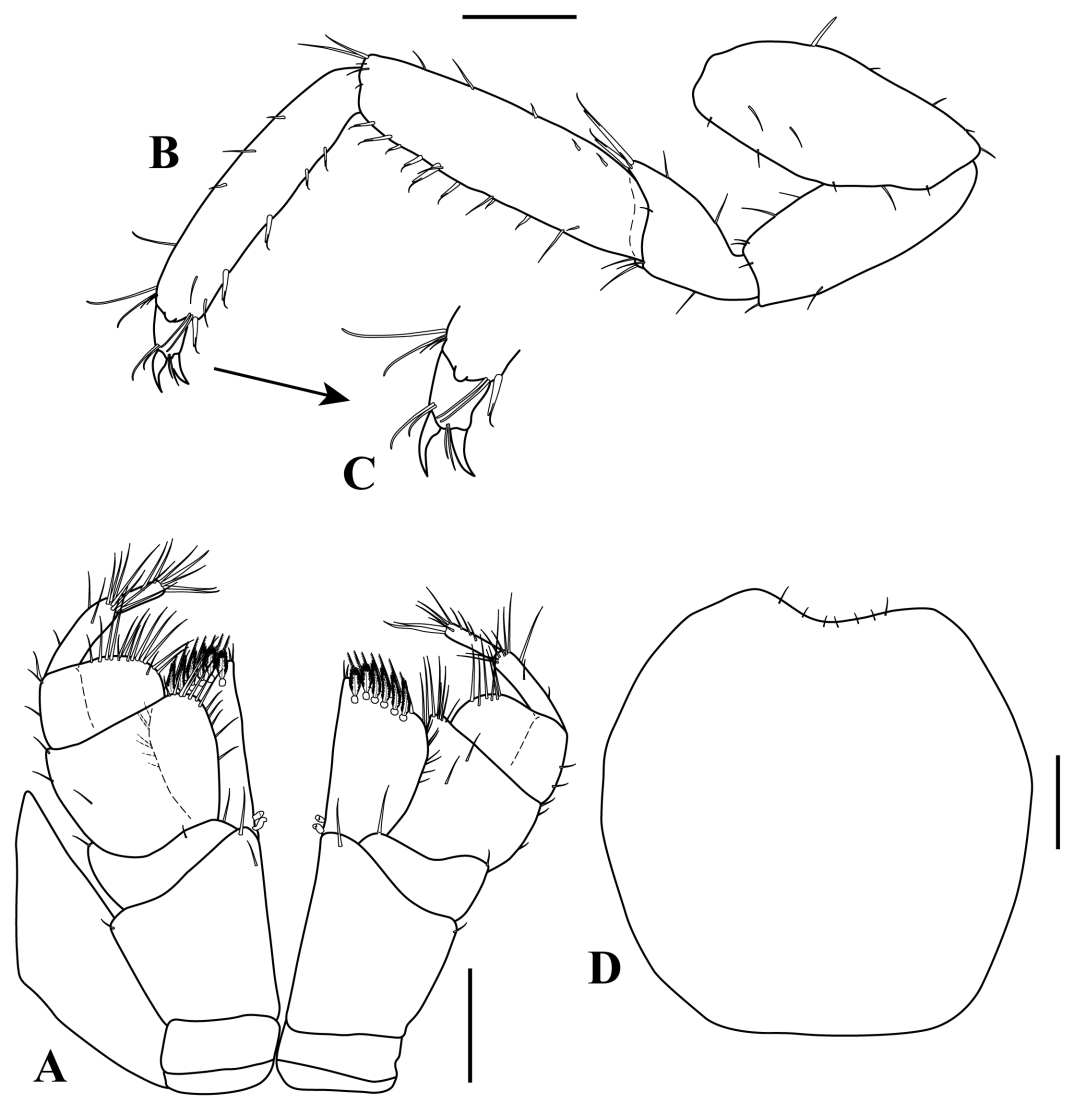
Ianiropsis cf. serricaudis from MCBs, ♀ with oostegites. **A** maxilliped **B** pereopod I **D** operculum. Scale bar: 0.1 mm.

*Maxilliped* (Fig. 12A). Maxillipedal palps cannot be observed on dorsal view, not passing well beyond the basal articles of the antenna. Basis, as long as wide. Endite, 2.0 times as long as wide, outer margin with 7–8 simple setae; inner proximal margin with two coupling hooks, sub-distal margin with seven (six on dorsal view and one on ventral view (not shown), respectively) fan setae (Fig. 8C), distal margin with 12 (seven and five (not shown), respectively) setulate setae (Fig. 8D), inner distal margin with two simple setae. Palp 2,4 times longer than basis: article 1, wider than long; article 2, wider than long, 1.3 times as long as article 1, inner distal margin with 7–8 simple setae of varying lengths; article 3, wider than long, 1.8 times as wide as long, slightly shorter than twice of article 2; inner distal margin with a row of 14–15 simple setae of varying lengths, outer margin with three (one on middle and two on distal) simple setae; article 4, 3.3 times as long as wide, with row of 6–7 simple setae on inner distal margin; article 5, slightly shorter than twice of article 4, 2.3 times as long as wide, with a row of 7–8 simple setae on inner margin.

*Pereopod I* (Fig. 12B–C). Shorter than male pereopod I, 0.48 times shorter than TL, Basis 2.0 times as long as wide; with six small simple setae along dorsal margin; with two simple seta on proximal-medial margin, and one robust and five simple setae along ventral margin. Ischium, 1.8 times as long as wide; with four simple setae along dorsal margin; with one simple seta on disto-medial margin; with two simple small setae along ventral margin. Merus, 1.5 times as long as wide, widest distally; disto-dorsal lobe with one simple and three robust distal setae, with two small setae along dorsal margin; with simple seta on disto-lateral margin; with four simple setae of varying lengths on ventral margin. Carpus, 3.4 times as long as wide; with five (three small) simple setae along dorsal margin and four disto-dorsal simple setae of unequal lengths; with simple seta on disto-lateral margin, with six simple setae and six robust setae along ventral margin. Propodus, 4.9 times as long as wide; with four simple setae along dorsal margin and cluster of four simple setae of varying lengths on distal dorsal margin (Fig. 12B–C); with simple seta on sub-distal lateral margin, with three (one sub-distal and two distal) simple setae and five robust setae along ventral margin, articular plate absent. Dactylus with two distal claws, with two simple setae on disto-medial margin between the claws, with two simple setae on disto-dorsal margin (Fig. 12B–C).

*Operculum* (Fig. 12C). As long as wide distal margin concave with small simple distal setae.

######### Ovigerous females.

Slightly smaller than males; mean TL 2.17 mm, ranges from 1.91 to 2.34 mm (Table 2). Antennule with 10–11 articles (Table 2). Antenna usually shorter than TL (Table 2); lengths of antennal articles 5–6 combined, shorter than half TL (Table 2). Maxillipedal palps cannot be observed on dorsal view, not passing well beyond the basal articles of the antenna.

######### Variations.

Ianiropsis cf. serricaudis from MCBs shows some degree of variations among the individuals as: 1) overall, the number of antennular articles varied between females and males; females have less articles than males (7–12 *versus* 9–14, respectively) (Table 2); 2) the total lengths of the antennal articles 5–6 combined is shorter than half TL of females and males (Table 2); 3) long maxillipedal palps can be easily observed in dorsal view on largest adult males (2.43–3.0 mm) (Table 2), but elongated maxillipedal palps can be observed also in males of less size (e.g. 2.17–2.22 mm); and 4) the number of denticles on the lateral margins of the pleotelson, ranged from 2 to 4; the three most common denticles combinations were 3–3 (32%), 4–4 (29%), and 3–4 (14%) (Table 2); sometimes the denticles are much less conspicuous.

######### Other material examined.

*Ianiropsis* sp. (USNM 99317): four ovigerous ♀♀, two ♂♂, station II-19 (29.6533 N, -80.38 W), off the coast of Florida, USA, depth 42 m, April-26-1953, identified by Bowman, Thomas E., Smithsonian Institution, National Museum of Natural History. *Ianiropsis* sp. (USNM 190327): one specimen (damaged, apparently ♀), (37.1017N – -74.5533 W), off the coast of Virginia, USA, depth 180 to 200 m, Sep-01-1976, identified by Virginia Institute of Marine Sciences.

## Discussion

The original description of *Ianiropsis serricaudis* given by Gurjanova (1936) was based on an apparently adult male with body length of about 3.0 mm that was not fully developed (e.g., pereopod I not elongate; p. 251, fig. 1). Conversely, I. cf. serricaudis male from MCBs with body length comparable to that of the original description shows a well-developed pereopod I, and males not fully developed (< 2.2 mm) from MCBs have a pereopod I that is not elongate. We corroborated that long maxillipedal palps can be easily observed in dorsal view on the largest adult males (G.D.F. Wilson, pers. comm.). Although, maxillipedal palps with the last three articles elongated are present in males less than 2.2 mm in body length, they are not complete or fully elongated as in adult males to be easily observed in dorsal view (Gurjanova 1936, Kussakin 1988, and this study, Table 2).

Recent studies, Hobbs et al. (2015), Marchini et al. (2016), and Ulman et al. (2017), suggest that *I. serricaudis* is well established along both coasts of the United States and the North-eastern Atlantic. The I. cf. serricaudis material collected and examined from MCBs is morphologically similar to the materials collected, reported, and illustrated (some characters) of *I. serricaudis* in USA (Hobbs et al. 2015) by having males with: (1) a pleotelson with three to four denticles on lateral margin, (2) adult males with elongated maxillipedal palp which can be seen on dorsal view, (3) dactylus of pereopod I with two claws, and (4) dactylus of pereopods II to VII with three claws. Nevertheless, there are subtle but significant differences between these materials such as: (1) lengths of the antennal articles 5–6 combined, shorter than half TL of males from MCBs *versus* longer than half TL of males from upper areas of North-east coast of USA; and (2) the lowest number of denticles on the lateral margins of the pleotelson registered on MCBs populations was two *versus* three from upper areas of North-east coast of USA. Clearly, there is a need to conduct a detailed revision of the original type material or specimens of *I. serricaudis* from the precise type locality (topotypes), and more detailed morphological and molecular analysis of the genus to determine the degree of similarity among the populations. To aid in identification of the species especially in the area, we have included a complementary detailed description of an adult male of I. cf. serricaudis from MCBs.

This is the first time that individuals attributable to I. cf. serricaudis have been reported from MCBs. This is also the first detailed illustration and description of an adult male of I. cf. serricaudis from MCBs in the western Atlantic. It is possible, however, that this new finding or possible new record is the result of a more intensive screening effort and careful examination of coastal marine invertebrates in MCBs. The small size of individuals belonging to the species and lack of taxonomic expertise might have led to them being overlooked or misidentified in samples collected previously from the bays by other investigators (Llansó et al. 2002, 2003, 2004, 2005, 2006; Llansó and Dew 2010; Llansó 2015). Furthermore, until a molecular study similar to the work on the isopod, *Asellus* (Verovnik et al. 2009) is conducted to determine if *I. serricaudis* populations from both coasts of the United States and worldwide are conspecifics, the possibility of the existence of undescribed species cannot be rule out.

Unfortunately, all the specimens from Florida examined in this study were in such a bad condition that it was difficult to conduct a detailed taxonomic identification. All of them had a pleotelson without denticles on lateral margin, implying that the specimens are not conspecific with *I. serricaudis*. The only specimen from off Virginia coast that was also examined in this study was equally in bad condition; nonetheless, it has a pleotelson with three denticles on lateral margin. Since this character has been reported in other species within the genus *Ianiropsis* and without the presence of an adult male, we cannot definitely state that the specimen belongs to *I. serricaudis*. In fact, the presence of this *Ianiropsis* specimen collected in 1976 indicates that this genus has been present on the East coast of the United States much earlier than the most recent records of this genus from Maine to New Jersey (Hobbs et al. 2015), and MCBs (this study). Besides, it is interesting that this specimen was collected within a depth range of 190 to 200 m. All members of *Ianiropsis* that have been reported around USA were collected in shallow waters (<10 m).

An illustrated key of males of *Ianiropsis* species belonging to the palpalis-group (Wilson and Wägele 1994) is presented. Wilson and Wägele (1994) established the term “palpalis-group” to include within the genus *Ianiropsis* all the males that have long maxillipedal palps, which can be observed on dorsal view, passing well beyond the basal articles of the antenna (Wilson and Wägele 1994; Doti and Wilson 2010).

### Key to species of *Ianiropsis* “*palpalis*-group”

**Table:** 

1	Pleotelson without denticles on each lateral margin (Fig. 13A)	***Ianiropsis palpalis* Barnard, 1914** [South Africa]
–	Pleotelson with two to four denticles on each lateral margin (Fig. 13B–D)	**2**
2	Pleotelson with two denticles on each lateral margin (Fig. 13B)	***Ianiropsis epilittoralis* Menzies, 1952** [North East Pacific: California]
–	Pleotelson with three to four denticles on each lateral margin (Fig. 13C–D)	***Ianiropsis serricaudis* Gurjanova, 1936 *sensu*Hobbs et al. 2015** [North West Atlantic: Maine to New Jersey]; and **Ianiropsis cf. serricaudis Gurjanova, 1936 *sensu* Morales-Núñez and Chigbu, this study** [North West Atlantic: Maryland)

**Figure 13.**
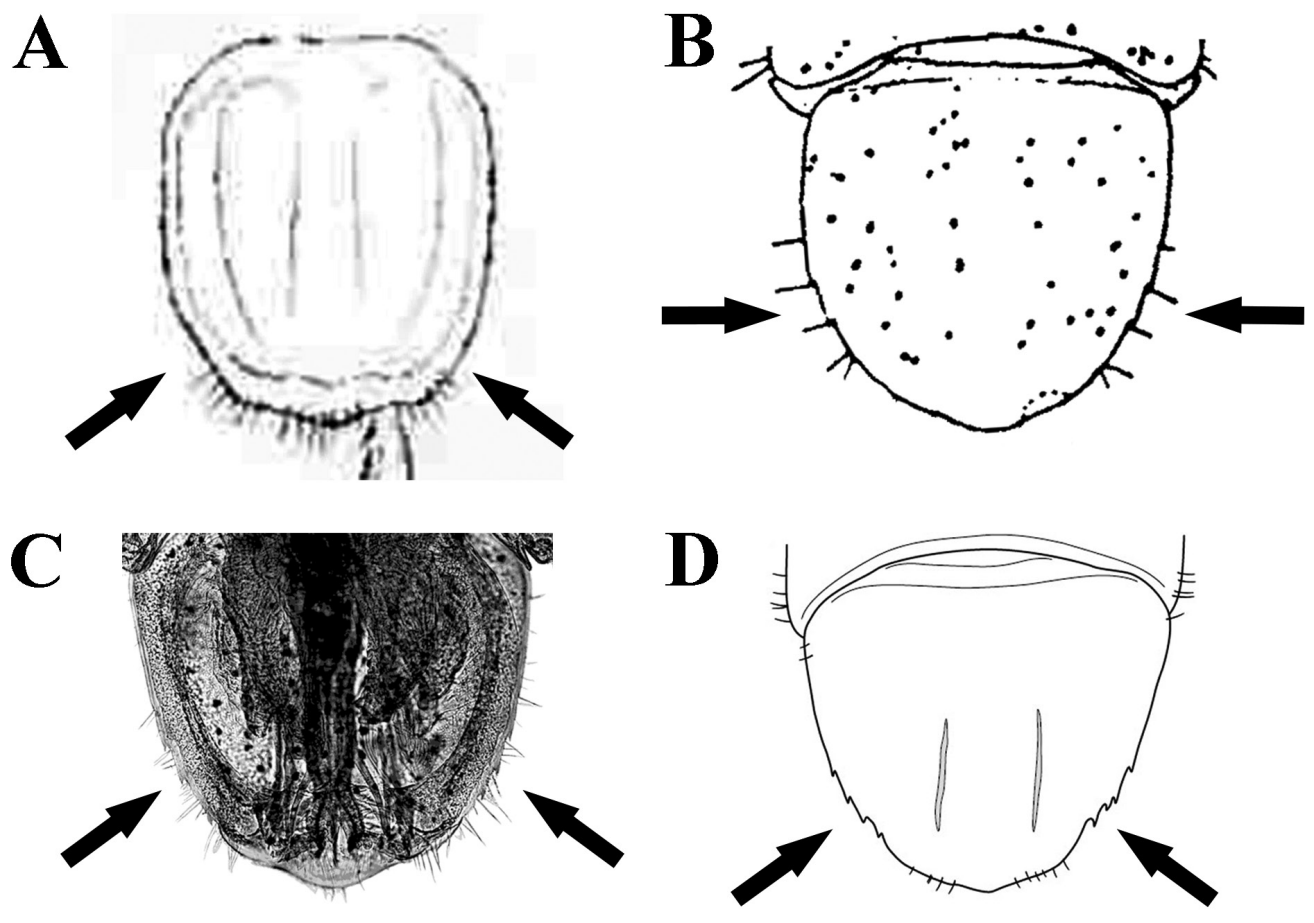
Denticles on lateral margin of pleotelson: **A** *Ianiropsis palpalis* **B** *I. epilittoralis* **C** *I. serricaudis* *sensu* (Hobbs et al. 2015) – Gulf of Maine to Barnegat Bay **D** I. cf. serricaudis this study – Maryland Coastal Bays. [Figures modified from (Barnard 1914; Menzies 1952; Hobbs et al. 2015), and this study]; not to scale.

### Habitat

Marine epibenthic, in coastal shallow waters (≤ 3.1 m); Ianiropsis cf. serricaudis was collected with a small mixture of macroalgae (e.g., *Gracilaria* sp. and *Ulva lactuca*). Physicochemical parameters of the surrounding waters were: temperature, 17.94 °C; salinity, 31.63 PSU; dissolved oxygen (mg l^–1^), 8.35; pH, 7.97, and Secchi depth transparency, 1.7 (m).

## Supplementary Material

XML Treatment for Ianiropsis cf. serricaudis
